# Evaluation of the Effectiveness of Feedback in a Remote Monitoring Home-Based Training System for Workers: A Medium-Scale Randomized Parallel-Group Controlled Trial

**DOI:** 10.3390/healthcare13162069

**Published:** 2025-08-21

**Authors:** Yasuhiro Suzuki, Hiroaki Kawamoto, Takaaki Matsuda, Hiroaki Suzuki, Hitoshi Shimano, Naoya Yahagi

**Affiliations:** 1 Institute of Systems and Information Engineering, University of Tsukuba, Tsukuba 305-8573, Ibaraki, Japan; kawamoto.hiroaki.gp@u.tsukuba.ac.jp; 2 Biomedical Science and Engineering Research Center, Hakodate Medical Association Nursing and Re-Habilitation Academy, Hakodate 040-0081, Hokkaido, Japan; 3 Division of Endocrinology and Metabolism, Department of Medicine, Jichi Medical University, Shimotsuke 329-0498, Tochigi, Japan; nyahagi-tky@umin.ac.jp; 4 Tsukuba Clinical Research and Development Organization (T-CReDO), University of Tsukuba, Tsukuba 305-8577, Ibaraki, Japan; matsuda.takaaki.dd@ms.hosp.tsukuba.ac.jp; 5 Department of Endocrinology and Metabolism, University of Tsukuba Hospital, Tsukuba 305-8575, Ibaraki, Japan; 6 Department of Food and Health Sciences, Institute of Human Life Sciences, Jissen Women’s University, Hino 191-8510, Tokyo, Japan; suzuki-hiroaki@jissen.ac.jp; 7 Department of Endocrinology and Metabolism, Institute of Medicine, University of Tsukuba, Tsukuba 305-8573, Ibaraki, Japan; hshimano@md.tsukuba.ac.jp; 8 Japan Agency for Medical Research and Development-Core Research for Evolutional Science and Technology (AMED-CREST), Chiyoda-ku 100-0004, Tokyo, Japan; 9 Life Science Center of Tsukuba Advanced Research Alliance (TARA), University of Tsukuba, Tsukuba 305-8577, Ibaraki, Japan; 10 International Institute for Integrative Sleep Medicine (WPI-IIIS), University of Tsukuba, Tsukuba 305-8577, Ibaraki, Japan

**Keywords:** SUKUBARA^®^, telerehabilitation, low-intensity resistance exercise, e-mail, unsupervised exercise, video-delivered exercise

## Abstract

**Background:** Maintaining long-term exercise adherence in occupational settings remains a challenge, particularly in remote or unsupervised environments. This study aimed to investigate the effect of individualized feedback on exercise adherence, body composition, and physical function during a remote home-based training intervention utilizing the video-based exercise system “SUKUBARA^®^”. **Methods:** In total, 66 care facility workers were randomly categorized into either a feedback (FB) group or a non-feedback (NF) group. Both groups performed a combined exercise program comprising low-load resistance training (slow squats) and balance exercises (one-leg standing time of closed eye) for approximately 15 min, thrice weekly over 12 weeks. The FB group received individualized feedback sheets visualizing total video play time (TT), exercise frequency, and interruptions, alongside reminder emails. The primary outcome was TT. Secondary outcomes included body composition measures (body weight, fat-free mass, and body fat mass rate) and one-leg standing time of opened eye. **Results:** The FB group demonstrated significantly greater TT, approximately 1.5 times that of the NF group, indicating enhanced exercise adherence. Moreover, significant improvements in fat-free mass and body fat mass rate were observed in the FB group. A significant correlation was identified between changes in TT and body composition parameters, suggesting TT as a valid proxy for exercise engagement. **Conclusions:** Individualized feedback within a remote monitoring home exercise program effectively improved exercise adherence and body composition among care workers. The “SUKUBARA^®^” system shows promise as a tool to support exercise continuity in occupational health and long-term care settings.

## 1. Introduction

In Japan, an increasing number of companies are adopting health management initiatives, with many implementing various workplace health promotion measures. To effectively implement such measures, organizational efforts, such as creating a supportive workplace environment, are essential. One way to improve the workplace environment is to provide exercise programs that encourage employees to develop exercise habits. Resistance exercise is an effective health exercise program that reportedly improves fat-free mass (FFM) and basal metabolism, prevents and manages chronic diseases, and improves activities of daily living and quality of life [1]. Furthermore, combining functional exercise with resistance exercise is reportedly more effective at preventing falls [2]. However, the proportion of working adults (aged 20–64 years) in Japan who engage in regular exercise remains low [3]. In addition, although the number of older adults in the workforce is increasing, their participation and compliance rates in exercise programs remain low [4]. Among different working populations, care facility staff are particularly vulnerable due to their high physical and emotional workloads, irregular hours, and limited opportunities to engage in structured physical activity during or after work. Despite their role in promoting the health and mobility of elderly residents, care workers’ own health needs, especially regarding musculoskeletal function and physical capacity, are often overlooked. Therefore, workplace-based interventions that are both accessible and adaptable are crucial for this group.

Barriers to exercise adherence include personal factors such as lack of motivation, boredom, fear of injury, and pre-existing health conditions [5]. Environmental and logistical challenges, such as limited access to exercise facilities, inadequate transportation, safety concerns, weather conditions, and financial constraints, further hinder participation [6,7,8,9]. To address these barriers, technology is increasingly being integrated into exercise programs, particularly in rehabilitation settings, to enhance participation and adherence (telerehabilitation) [10].

Among these technological innovations, video-based exercise demonstrations have recently gained attention as a means of instructing exercise habits. Compared to text-based instructions, videos reportedly offer increased comprehension, engagement, and adherence by providing clear visual demonstrations, verbal instructions, and often motivational background music [11,12,13]. Therefore, we developed a novel video-based exercise adherence system, called SUKUBARA^®^, which delivers structured training videos via YouTube and records total video playtime (TT) as an indirect indicator of exercise implementation. The system allows administrators to remotely monitor participants’ engagement and flexibly adapt exercise protocols by uploading customized videos. In a previous study, SUKUBARA^®^ was used to implement a 12-week resistance and balance training intervention among hospital staff, resulting in significant improvements in fat-free mass (FFM), knee extension strength, and one-leg standing time (OLS), compared to a control group. Moreover, a positive correlation between TT and training outcomes indicated that video viewing time could serve as a valid proxy for exercise adherence [14,15]. However, this prior study did not investigate the role of feedback in promoting sustained engagement. Feedback, such as visualizing one’s own progress, is considered a critical factor in ensuring long-term behavior change, particularly in self-directed exercise [16]. In telerehabilitation and home-based programs, this factor becomes even more important due to limited face-to-face supervision.

The present study aimed to examine whether integrating a feedback system within SUKUBARA^®^ would improve participants’ exercise adherence (i.e., TT) and whether this increased adherence would lead to measurable changes in body composition and physical function. We further assessed the relationship between TT and individual improvement metrics, offering insights into how feedback mechanisms can optimize remote exercise interventions.

## 2. Materials and Methods

### 2.1. Participants

The study targeted 120 workers from seven nursing care facilities operated by DOCTOR EYE’S CO, LTD, Sapporo, Japan between August and October 2024. Recruitment flyers were posted and distributed at each facility, and individuals who applied were enrolled sequentially according to the inclusion and exclusion criteria. The inclusion criteria were as follows: (1) aged between 20 and 75 years; (2) independent walking and daily living activities; (3) ownership of a smartphone with unrestricted YouTube access; and (4) a smartphone data plan (approximately 5 GB) sufficient for approximately 15 min of daily video streaming (or access to a home Wi-Fi network). Exclusion criteria included people whose daily living activities and exercise have been restricted by a doctor. Participants were only responsible for communication fees when participating in this study. As compensation, a JPY 1000 QUO card was provided upon obtaining written informed consent. In addition, no refund was requested from participants who withdrew during the study.

Physical fitness tests were conducted at participant workplaces during the pretest (between October and November 2024) and post-test (between January and February 2025). The study was designed as a parallel group comparative study, and participants were randomly assigned to either a Feedback (FB) or a No Feedback (NF) group. Participants were randomized into the two groups in the order of their registration using the blockrand function [17] in the statistical software R (R version 4.3.2) [18]. A designated study coordinator (other than the person in charge of the study) managed the registration and sequentially issued allocation slips to participants upon enrollment. Although participants were not blinded, the study was designed as a single-arm, blinded study because the individuals administering the fitness tests could not distinguish between individual participant groups. Both groups were instructed not to develop new exercise habits during the study period.

As this study is a new attempt, a sample size of 90 people was set from the perspective of feasibility, assuming that approximately 70% of the 120 workers would apply. All participants received verbal and written explanations of the purpose and procedures of the study, along with the fact that they could withdraw at any time without disadvantages. Written informed consent forms were obtained from all participants before the study. The study protocol was approved by the Medical Ethics Committee of the Jichi Medical University Hospital (approval number 24-043), and the study has been registered in the clinical trial database (UMIN000055297).

### 2.2. Remote Monitoring At-Home Training System (SUKUBARA^®^)

This system is characterized by its use of web technology to record and manage individual video viewing status in real-time. By combining flexible data management by Firestore (Google LLC, Mountain View, CA, USA) and an authentication infrastructure by Clerk, administrators can accurately grasp the viewing status and provide appropriate guidance and intervention, even remotely. For security, access is authenticated through Clerk, and all communications are encrypted with HTTPS. In addition, Firestore access is restricted to the server side, preventing vulnerabilities caused by incorrect security rule settings.

A training video is created based on the designated exercise program and uploaded to a dedicated YouTube channel. The system retrieves the playback URL of the video, converting it into a unique QR code linked to an ID number for each participant (enabling access to identical content of target participants), and generates a training sheet. The training sheet is distributed to each individual on paper. Participants play the video by scanning the QR code using the camera function of a smartphone or tablet device during training, while simultaneously carrying out the exercise program, and the viewing time for video playback is recorded. The training video has a duration of 14 min and 30 s. Although the source video is publicly accessible, the program video can only be assessed via the training sheets, allowing administrators to remotely monitor participant activities. In light of these functions, we designated the system as a “remote monitoring at-home training system” and named it SUKUBARA^®^ (Advanced Research Initiative for Human High Performance, University of Tsukuba, Tsukuba, Japan).

### 2.3. Interventions

In 2024, we reported the details of a composite exercise program using SUKUBARA^®^ [15]. Previous studies have shown that resistance exercise alone is less effective and that combining it with walking and applied movement practice can produce more substantial effects [2]. Based on the composite effect of exercise, we designed a 15 min program comprising two low-load resistance exercises [19]: slow squats and balance exercise OLS with closed eyes (10 slow squats × 3 sets, OLS 60 s × 6 sets).

**Figure 1:**
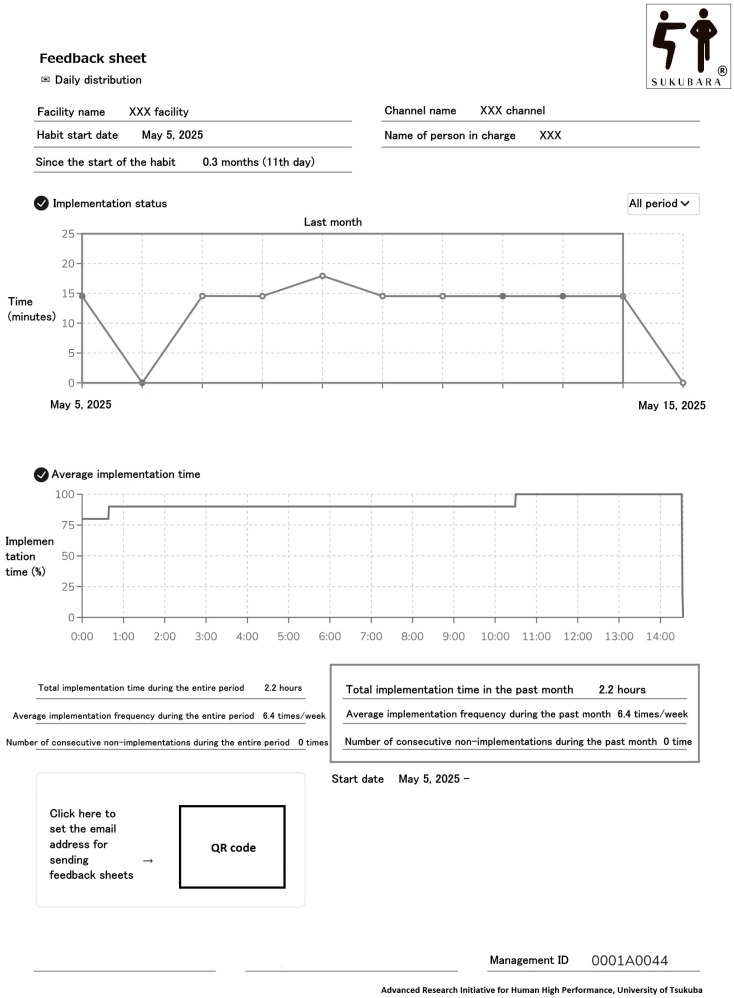
Example of a feedback sheet from the SUKUBARA^®^ system.

The FB group was given a feedback sheet (Figure 1) in addition to the exercise program, while the NF group only performed the exercise program. The feedback sheet included the start date of the exercise, the period from when the exercise started to the present, a plot graph of total video playtime, exercise frequency (TT corrected by the duration of the exercise program; times/week), and the number of interruptions (a break was defined as not playing the video for one consecutive week; times). Feedback sheets were sent daily by email. For the FB group, if the assigned video was not played for 7 consecutive days, an alert email was sent on the 8th day to warn participants. The alert email read, “Thank you for your efforts in maintaining your daily habits. You may not have maintained your habits for the past week. Please do not let your past habits go to waste.” All participants received initial in-person instructions from a collaborator at their respective facilities, including how to watch videos and train using their smartphones, and the training was conducted under supervision at the facility only for the first time. During the initial interview, which included the provision of exercise instruction and guidance on how to operate the system, participants were verbally informed that the research director would monitor their exercise status and any interruptions on a daily basis. The program was to be conducted at least thrice weekly for 12 weeks, and participants were encouraged to train daily if possible.

### 2.4. Measurements

The primary outcome measure in this study was TT of each participant; secondary outcomes included changes in body composition and OLS of opened eye. TT was expressed as the number of hours (hours/12 weeks) immediately after the end of the intervention period. Body composition was assessed by weight (kg), FFM (kg), and body fat mass rate (%) and was measured using bioelectrical impedance analysis. A home body composition analyzer (InBody H20N; InBody Japan Inc., Tokyo, Japan) was used to evaluate body composition. This device employs multi-frequency bioelectrical impedance analysis (BIA) technology, similar to InBody’s commercial devices, and is characterized by its ability to easily and non-invasively measure major body composition indicators such as weight, FFM, and body fat mass rate. Although the InBody H20N has not yet been approved as a medical device, the company’s other commercial BIA device (InBody 720; InBody Japan Inc., Tokyo, Japan) reportedly shows a high correlation with dual-energy X-ray absorptiometry (correlation coefficient: muscle mass 0.969, fat mass 0.935) [20]. Notably, the InBody H20N is cheaper and lighter than commercial devices, and can be installed in nursing care facilities with limited installation space, consistent with the survey design of this study, which involved comprehensive deployment in multiple stores. All measurements were performed under standardized conditions, and every effort was made to minimize errors. The measurement time was fixed at 9:00 AM (following an overnight fast from 9:00 PM) to control for environmental factors, such as meal intake and time of day. The measurements of OLS of opened eye (s) were conducted in accordance with prior research [15]. These items were measured before and after the 12-week intervention by the facility directors, who served as the co-researchers at each facility. To ensure inter-rater reliability in OLS measurements, a manual with clear endpoints was distributed (e.g., using a stopwatch, setting the upper limit to 120 s, ending when the raised foot touches the floor, etc.). Previous reports have shown that inter-rater reliability in OLS measurements is good [21].

### 2.5. Statistical Analysis

For all evaluation items, Shapiro–Wilk normality tests were performed to assess the normality of data distribution, and appropriate statistical methods were selected. Variables with normal and non-normal distributions were presented as the mean ± standard deviation and median (interquartile interval 25th percentile, 75th percentile), respectively. Baseline between-group comparisons, including age, sex, height, weight, body mass index, lifestyle factors (exercise habits, drinking habits, community activities, and driving status), chronic illness (diabetes, dyslipidemia or hypertension, chronic bronchial asthma, and lower back pain), body composition (FFM and body fat mass rate), and OLS before intervention were performed using unpaired *t*-tests, Mann–Whitney U tests, and chi-square tests.

To compare the between-group changes in TT, body composition, and OLS during the intervention, independent *t*-tests (for normally distributed variables) or Mann–Whitney U tests (for non-normally distributed variables) were used. For normally distributed data, a two-way analysis of variance (ANOVA) with group (FB vs. NF) and time (pre- vs. post-intervention) as factors was used to assess main effects and interactions. In addition, Bonferroni correction was performed after two-way ANOVA. For non-normally distributed data, the Wilcoxon signed-rank test was used for comparison. For the primary outcome, TT, if normally distributed, the mean and standard deviation of each group were calculated, and the mean ratio and its 95% confidence interval were estimated by logarithmic transformation. Cohen’s d was calculated as the effect size for the mean difference between groups. Standard deviations were combined using pooled standard deviations.

If a difference in the change in a factor was observed between groups, the correlation with TT was examined. Partial correlation analysis was adjusted for age and sex, and Spearman’s rank correlation coefficients were conducted for normally and non-normally distributed variables. Statistical analysis was performed using SPSS version 24.0 (IBM SPSS Statistics; IBM Japan, Ltd., Tokyo, Japan), and the statistical significance level was set at 5%.

## 3. Results

**Figure 2:**
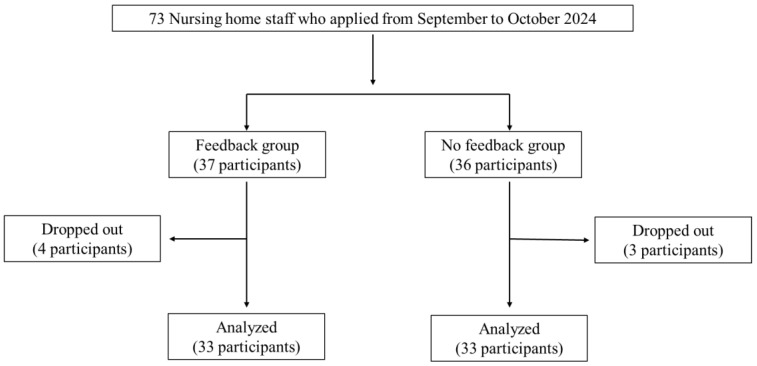
Flowchart study participant selection.

This study initially aimed to recruit 90 participants; however, only 73 applied. Of the 73 participants, 37 were randomly assigned to the FB group and 36 to the NF group. Seven participants dropped out (six did not attend the final evaluation, and one withdrew); however, no adverse events were reported during the intervention. The final analysis included 33 participants each in the FB and NF groups (follow-up rate 90%; Figure 2). The final breakdown of the subjects by occupation was as follows: 51 (77%) caregivers, 9 (14%) medical professionals or functional training instructors, and 6 (9%) administrative or managerial staff. A total of 70 alert emails were sent to the FB group.

**Figure 3:**
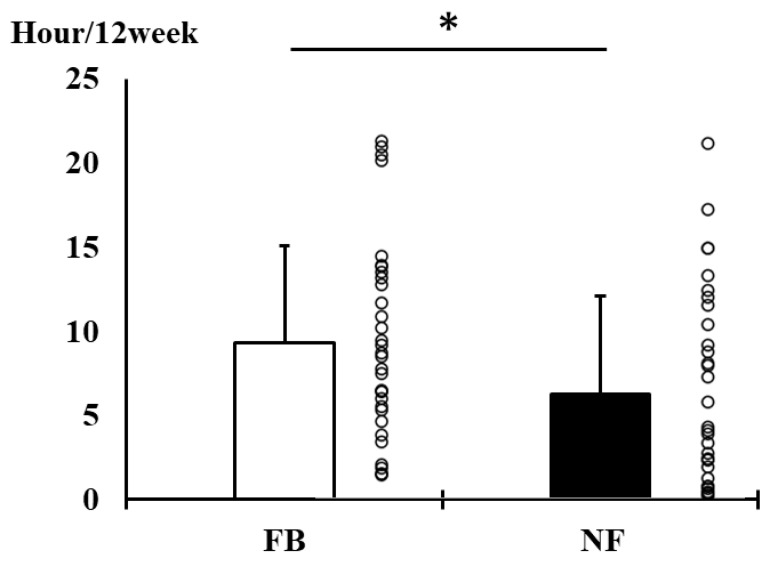
Comparison of total video play time between groups. FB, Feedback group; NF, No feedback group * < 0.05.

Table 1 summarizes the basic participant characteristics, lifestyle factors, chronic illness, body composition, and OLS. No significant between-group differences were observed in any of the parameters. Figure 3 shows a comparison of TT during the intervention period between groups (FB vs. NF). A significant between-group difference was observed in TT (9.3 ± 5.8 vs. 6.3 ± 5.8 h/12 week, *p* = 0.044). The ratio of mean TT in both groups was 1.48 times (95% confidence interval: 1.01–2.16), and a moderate effect (Cohen’s d: 0.52) was observed. Table 2 (body composition) and Table 3 (OLS) show between- and within-group comparisons of changes pre- and post-intervention. In Table 2, all factors showed no change over time during the intervention period; however, significant between-group differences were observed in changes in FFM (0.4 ± 1.3 vs. −0.2 ± 0.7 kg, *p* = 0.041) and body fat mass rate (−1.0 ± 3.1 vs. 0.5 ± 2.0%, *p* = 0.011). No significant group × time interactions were observed. Table 3 shows the OLS results. Because OLS was not normally distributed, interactions were not examined, and analyses were performed separately within and between groups. During the intervention, only the FB group exhibited a significant increase (0 s, *p* = 0.021); however, no significant between-group differences were observed.

**Figure 4:**
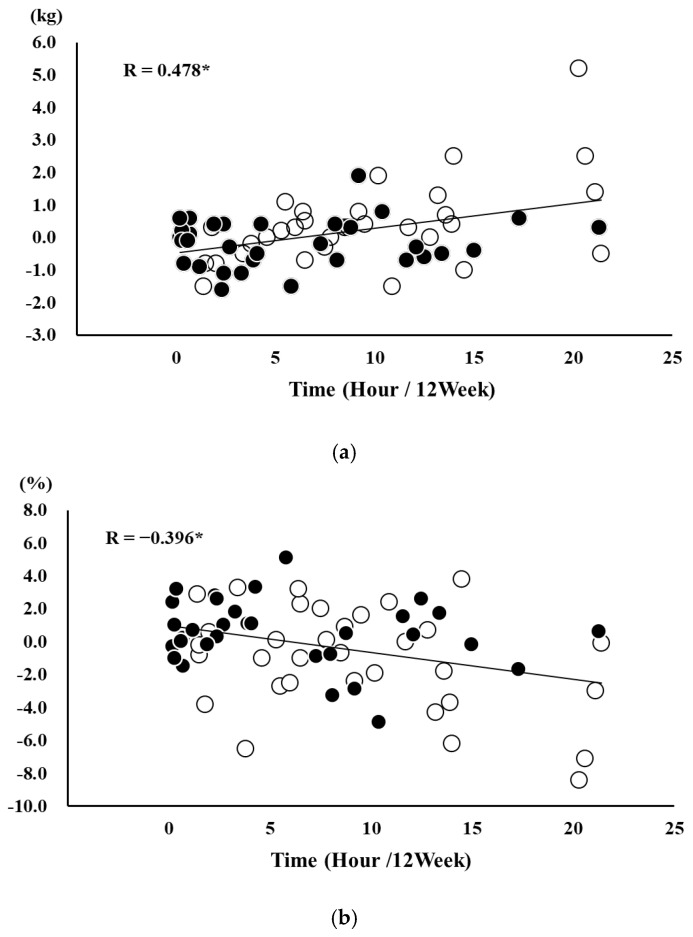
(**a**) Relationship between change in fat-free mass and total video playtime. (**b**) Relationship between change in body fat rate and total video playtime ○: Feedback group. ●: No feedback group. * < 0.001.

Because the changes in TT, FFM, and body fat rate showed normal distributions, partial correlation analysis adjusted for age and sex was performed, and the results showed significant correlations between TT and changes in FFM and body fat rate (R = 0.478, *p* < 0.001; Figure 4a) (R = −0.396, *p* < 0.001; Figure 4b).

## 4. Discussion

This study investigated the effects of a feedback system using the SUKUBARA^®^ remote monitoring home training system over a 12-week period, targeting workers at a long-term care insurance facility. Results showed that TT during the intervention was significantly higher in the FB group than in the NF group. When the TT was translated into exercise frequency, the FB group averaged 3.2 ± 2.0 sessions per week, compared to 2.2 ± 2.0 sessions in the NF group. This suggests that providing daily feedback via email based on viewing records increased exercise frequency by approximately 1.5 times. These results support the study’s hypothesis, with a particularly important finding being that exercise frequency increased despite the use of a simple, automated email feedback system. This finding suggests that the feedback system using SUKUBARA^®^ is cost-effective. Future studies should expand the target population to include individuals with a broader range of occupations and ages to improve the generalizability of findings. Additionally, it would be beneficial to explore the long-term effects of the system and evaluate its cost-effectiveness in real-world settings, particularly in community health promotion or preventive care programs.

Other studies have shown the effect of improving exercise habits or physical activity levels at home [22,23,24]. In a prior study of subjects with knee osteoarthritis and obesity (median age: 62 years; 67% female; approximately 60% employed), one study reported that feedback using automated semi-interactive short messages improved adherence to home exercise in an unsupervised environment [24], and the results of this study were similar. However, the content of the feedback in this study was not primarily verbal, such as encouragement or appreciation, but was focused on visualizing the status of exercise implementation. One prior study targeting community-dwelling individuals with type 2 diabetes who had low daily activity levels (median age: 64 years; average daily step count: approximately 3550 steps/day) reported that feedback visualizing their physical activity levels improved both step count and the duration of moderate-to-vigorous intensity activity [23]. These findings suggest that visualization of implementation records also affects adherence to resistance exercise at home.

One of the key contributions of this study is the possibility of further improving the quality of telerehabilitation. Telerehabilitation is defined as “the application of information and communication technology to provide rehabilitation treatment to people in remote locations” [25]. Although the participants of this study were not patients but workers in need of exercise training, the intervention can be viewed as a form of preventive telerehabilitation aimed at reducing future health risks, such as illness or workplace falls. In recent years, technology-based exercise interventions have expanded to community and residential environments, providing accessible and affordable ways to promote physical activity [26]. Advancements in digital technologies, such as computers, tablets, and smartphones, have made it increasingly convenient and flexible to deliver exercise programs [26,27]. Furthermore, the widespread use of the Internet and smartphones has further improved the accessibility of video-based exercise, enabling participation from remote locations at a relatively low cost. SUKUBARA^®^, used in this study, is a system that has fully benefited from these technological developments and device environments, especially video viewing and feedback systems. Therefore, the SUKUBARA^®^ system, when combined with a feedback intervention, appears to be an effective approach for promoting exercise among workers in more remote locations, those working from home under unsupervised conditions, and older workers, and is thought to contribute to corporate health management.

In this study, the changes in FFM and body fat mass rate in the FB group were significantly increased (+1.7% compared to pre) and decreased (−3.0% compared to pre) compared to the NF group, respectively. This result may be attributed to the higher frequency of exercise, as reflected by TT. In fact, TT was positively and negatively correlated with the changes in FFM and body fat mass rate, respectively, supporting our hypothesis. A meta-analysis reported that resistance exercise for older adults is more effective when performed more frequently, leading to significant improvements in muscle strength and muscle quality in older patients with sarcopenia [28]. In addition, our previous report also found that TT was significantly correlated with both knee extension strength and single-leg standing time when intervention was performed using SUKUBARA^®^ [14]. In this study, it is believed that the frequency of exercise at home influenced the FFM and body fat mass rate, and similar effects were shown.

Another important finding is that TT serves as a reliable indicator of actual exercise engagement. If the participants made many mistakes, such as not exercising while watching the video or not exercising seriously, the effects on body composition and the correlation between TT and body composition would not have been observed, which means that the SUKUBARA^®^ maintained the quality of exercise with a certain degree of accuracy. This finding is advanced for unsupervised exercise programs and shows that it is possible to monitor exercise status at home using TT, which can be easily collected.

### Limitations

This study has five limiting factors. Firstly, the sample size for this study was set based on feasibility considerations, rather than on power calculations based on effect sizes for the primary endpoint. While this is a practical approach, it may limit the statistical power to detect clinically meaningful differences. As such, we cannot rule out the possibility that small clinical effects for each endpoint may not have been detected in the selected sample size. In future studies, the sample size will be reviewed, the intervention study will be reconducted, and the results obtained will be interpreted by significance testing, allowing for final verification. Second, although this study observed changes in body composition, we were unable to draw definitive conclusions regarding the effects of training on these changes. This is primarily due to the use of a non-medically certified, consumer-grade body composition scale, whose accuracy and reliability cannot be guaranteed. Such devices are more susceptible to measurement error and may not accurately detect subtle physiological changes. Therefore, the findings related to body composition must be interpreted with caution. Future studies should employ medically approved instruments or gold-standard methods such as DEX to ensure the validity of the body composition measurements. Third, this study did not show any intervention effect on balance items. In our pilot study of hospital staff, we found that balance ability (OLS of closed eye) improved along with FFM improvement and knee extension strength improvement in non-older adults [14], which is different from the results of this study. A possible reason is that the OLS of open eye was used to evaluate balance ability in this study, which may have been too easy for the participants; therefore, there may have been a ceiling effect on the measurement results. Consequently, the median OLS time with eyes open reached a maximum of 120 s for both groups at baseline. However, there was some variation in the FB group at baseline, with the 25% lower limit being a low value of 49 s. This was thought to be due to chance variation, and since the FB group had a certain amount of “room for improvement”, it is possible that significant improvement was observed both before and after intervention. However, there was a ceiling effect in the OLS for both groups, and the median change was 0 s, indicating that the effect size was small, and no difference between the groups was observed. In future studies, the effect of improving balance ability by this intervention, if appropriate evaluation indexes are used, should be considered. Fourth, the intervention effect indicators in this study lacked a perspective that focused on the workforce. For example, if an increase in workers’ FFM led to a decrease in falls during work, or a decrease in workers quitting their jobs, a clear solution would be obtained. By implementing the SUKUBARA^®^ intervention on a large scale among local workers (including older adults) and evaluating it from a medium- to long-term perspective, the true effects could be verified. Finally, the results of this study were collected from participants from a specific age group and occupational setting, which limits the generalizability of the findings. However, similar work environments, such as care facilities, are not uncommon, and the present findings may still have practical applicability. In particular, this system may be of value in rural, car-dependent communities where opportunities for physical activity tend to be limited.

## 5. Conclusions

A 12-week intervention using SUKUBARA^®^ (resistance exercise + balance exercise) with a feedback system was conducted on workers at a long-term care insurance facility, and the TT of the exercise program video increased by about 1.5 times, and an increase in skeletal muscle mass and a decrease in body fat were observed. The findings suggest that this intervention promotes exercise habits in workers and can also be expected to improve body composition.

## Figures and Tables

**Table 1:** Participant characteristics.

	FB *n* = 33	NF *n* = 33	*p*
Age (year)	56 (40, 63)	58 (46, 66)	0.551
Female sex, *n* (%)	22 (66.7)	25 (75.8)	0.418
Height (m)	1.61 (1.55, 1.65)	1.60 (1.56, 1.64)	0.903
Body weight (kg)	63.5 ± 12.1	61.2 ± 12.4	0.852
Body mass index (kg/m^2^)	24.0 ± 3.5	23.6 ± 3.9	0.641
Exercise habits, *n* (%)	21 (63.6)	19 (57.6)	0.617
Drinking habits, *n* (%)	18 (54.5)	18 (54.5)	1.000
Community activities, *n* (%)	25 (75.8)	25 (75.8)	1.000
Currently driving, *n* (%)	32 (97)	32 (97)	1.000
Chronic illness, *n* (%)	15 (45.5)	11 (42.4)	
Diabetes, *n* (%)	1 (3)	1 (3)	1.000
Dyslipidemia or hypertension, *n* (%)	9 (27.3)	11 (33.3)	1.000
Chronic bronchial asthma, *n* (%)	2 (6.1)	1 (3)	1.000
Lower back pain, *n* (%)	3 (9.1)	1 (3)	1.000
Body composition			
Fat-free mass (kg)	23.3 ± 5.6	22.5 ± 4.5	0.540
Body fat mass rate (%)	32.8 ± 7.1	33.5 ± 7.9	0.724
Balance capability			
One-leg standing time of opened eye (s)	120 (49, 120)	120 (110, 120)	0.557

Data are presented as the mean ± SD or median (25th, 75th). FB, Feedback group; NF, No feedback group; SD, standard deviation.

**Table 2:** Body composition comparison of participants pre- and post-intervention and between groups.

	Group	Baseline	12 Weeks	*p* for Time	Change Between Baseline and 12 Weeks	*p* for Group	*p* for Group × Time
Body composition							
Body weight (kg)	FB	63.5 ± 12.1	63.6 ± 12.1	0.981	0.1 ± 1.6	0.638	0.963
	NF	62.9 ± 11.7	62.8 ± 11.9	0.967	−0.1 ± 1.7		
Fat-free mass (kg)	FB	23.3 ± 5.6	23.7 ± 6.0	0.772	0.4 ± 1.3	0.041 *	0.768
	NF	22.5 ± 4.5	22.4 ± 4.6	0.899	−0.2 ± 0.7		
Body fat mass rate (%)	FB	32.8 ± 7.1	31.8 ± 7.9	0.597	−1.0 ± 3.1	0.011 *	0.583
	NF	33.5 ± 7.9	34.0 ± 8.3	0.805	0.5 ± 2.0		

Data are presented as the mean ± SD. FB, Feedback group; NF, No feedback group; SD, standard deviation. * < 0.05.

**Table 3:** Balance capability comparison of participants pre- and post-intervention and between groups.

	Group	Baseline	12 Weeks	*p* for Time	Change Between Baseline and 12 Weeks	*p* for Group
Balance capability						
One-leg standing time of opened eye (s)	FB	120 (49, 120)	120 (120, 120)	0.021 *	0 (0, 3)	0.925
	NF	120 (110, 120)	120 (120, 120)	0.245	0 (0, 10)	

Data are presented as the median (25th, 75th). FB, Feedback group; NF, No feedback group. * < 0.05.

## Data Availability

The data used to support the findings of this study are available from the corresponding author upon request. The data are not publicly available because they contain information that can compromise the privacy of the research participants.

## Abbreviations

The following abbreviations are used in this manuscript:

FFM	Fat-free mass
OLS	One-leg standing time
TT	Total video play time
BIA	Bioelectrical impedance analysis
FB	Feedback group
NF	Non-feedback group
